# The effectiveness of behavioral economics-informed interventions on physician behavioral change: A systematic literature review

**DOI:** 10.1371/journal.pone.0234149

**Published:** 2020-06-04

**Authors:** Sophie Y. Wang, Oliver Groene

**Affiliations:** 1 OptiMedis AG, Hamburg, Germany; 2 Hamburg Center for Health Economics, Hamburg, Germany; 3 London School of Hygiene & Tropical Medicine, London, England, United Kingdom; University of Milan, ITALY

## Abstract

**Objective:**

Interventions informed by behavioral economics have the potential to change behaviors governed by underlying cognitive biases. This has been explored extensively for various use in healthcare including changing patient behavior and, more recently, physician behavior. We aimed to systematically review the literature on the use and effectiveness of behavioral economics-informed interventions in changing physician behavior.

**Method:**

We searched Medline, Cochrane Library, EBM Reviews, PsychINFO, EconLit, Business Source Complete and Web of Science for peer-reviewed studies published in English that examined the effectiveness of behavioral economics-informed interventions on physician behavioral change. We included studies of physicians in all care settings and specialties and all types of objectively measured behavioral outcomes. The reporting quality of included studies was appraised using the Effective Public Health Practice Project tool.

**Results:**

We screened 6,439 studies and included 17 studies that met our criteria, involving at least 9,834 physicians. The majority of studies were conducted in the United States, published between 2014 and 2018, and were in the patient safety and quality domain. Reporting quality of included studies included strong (n = 7), moderate (n = 6) and weak (n = 4). Changing default settings and providing social reference points were the most widely studied interventions, with these studies consistently demonstrating their effectiveness in changing physician behavior despite differences in implementation methods among studies. Prescribing behavior was most frequently targeted in included studies, with consistent effectiveness of studied interventions.

**Conclusion:**

Changing default settings and providing social reference points were the most frequently studied and consistently effective interventions in changing physician behavior towards guideline-concordant practices. Additional theory-informed research is needed to better understand the mechanisms underlying the effectiveness of these interventions to guide implementation.

## Introduction

The integration of research evidence into routine clinical practice to ensure safe and effective care for patients and reduce unnecessary expenditures has been a long-standing challenge [1,2]Studies in the United States, Netherlands, and Canada have shown that 30% to 40% of patients do not receive guideline-concordant care, and that more than 20% of care provided is unnecessary or potentially harmful [3,4]. Accordingly, given physicians’ role as key decision makers in healthcare, an increased focus on physician behavioral change has emerged [4–6].

Behavioral change is complex as it is influenced by a wide range of intrinsic and extrinsic factors such as an individual’s motivation, skills and knowledge as well as the social and physical environment [7]. Traditional behavioral change approaches to better align clinical practice with research evidence have mainly focused on improving access to information, such as guideline dissemination and education seminars. These methods are based on conventional economic theory and presume that physicians are perfectly rational decision-makers. However, research in behavioral economics, an evolving field rooted in economics and psychology, finds that humans have a “predictable” bounded rationality and rarely behave as the utility maximizers conventional economics theory would predict [8].

Physicians make numerous complex decisions on diagnostic and treatment plans daily, often with limited information and under time pressure [9]. When making high-volume of decisions under conditions of uncertainty, the decision-making process may be guided by environmental cues and heuristics [9,10]. While these strategies can be adaptive in clinical environments where high volume rapid decision-making is required, they can also be vulnerable to cognitive biases [11]. Systematic cognitive biases may affect the decisions of physicians thereby distorting probability estimation and impairing information synthesis [12]. Such biases may underlie why physicians sometimes continue to deliver care that robust evidence has shown to be of low value [13].

Interventions that aim to counteract the adverse effects of these cognitive biases are receiving growing attention, and have been applied in a variety of fields including energy, finance, taxation, and environmental studies [14,15]. In healthcare, behavioral economics-informed interventions have primarily been applied to changing patient behaviors, such as improving dietary choices [16–18], increasing preventative health screening participation [19,20] and increasing vaccination rates [21]. There has also been an increasing interest in using such interventions for provider behavioral change, but a systematic synthesis of the empirical evidence is lacking. Therefore, we systematically evaluated the literature to date on the use and effectiveness of behavioral economics-informed interventions in changing physician behavior. We focused specifically on physicians given their professional autonomy and their key decision-making role in team-based care models.

## Methods

We conducted and report the systematic review in accordance with the Preferred Reporting Items for Systematic Reviews and Meta-Analyses Protocols (PRISMA-P) guidelines [22]. We searched the International Prospective Register of Systematic Reviews (PROSPERO) prior to conducting this systematic literature review to ensure that no similar reviews have been conducted and registered the review protocol on PROSPERO in June 2019 (ID: CRD42019134956).

### Definition of behavioral economics-informed intervention

A “nudge” is defined as an intervention that predictably changes human behavior without limiting free choice or changing financial incentives significantly [23]. In the context of changing physician behavior, an example might be setting generic medications as the default when trying to decrease branded prescribing by physicians with the assumption that physicians prescribe more branded drugs due to an underlying salience bias. In this systematic review, we chose the term “behavioral economics-informed intervention” to broaden the concept, and we define this as an intervention designed to change behavior within a decision context by counteracting an underlying cognitive bias [24–26].

### Search strategy

We first conducted a broad and exploratory search to identify key terms under the umbrella term of behavioral economics-informed intervention; this took place in November 2018. We then conducted a second search in Medline using a combination of MeSH terms and free text terms within each of the population and intervention categories in the title and abstract search field (Table 1). We adapted and optimized the syntax individually for other databases. We searched databases from both medical, economics, and business administration fields including Medline, Cochrane Library, EBM Reviews, PsychINFO, EconLit, Business Source Complete and Web of Science. Our search included articles from inception until September 2019 to ensure we captured the evolution of this growing field. We then augmented the search using references from included articles and relevant reviews.

**Table 1:** Search terms used for Medline (OVID) search.

	Free Text Terms	MeSH Terms
Population	Physician*	Physicians
	Healthcare provider*	Health personnel
	Health care provider*	General Practitioners
	Doctor*	Family Physicians
	General practitioner*	
	Family doctor*	
Intervention	Behavio?ral economic*	
	Asymmetric paternalism	
	Nudg*	
	Choice Architect*	
	Reframe	
	Loss aversion	
	Endowment	
	Prospect theory	
	Feedback	
	Peer comparison	
	Social comparison	
	Social norm	
	Default	
	Status quo	
	Active choice	
	Prompted choice	
	Accountable justification	
	Suggested alternative	
	Mental accounting	
	Allocation bias	
	Reminders	
	Salience	
	Self control	
	Commit*	
	Precommitment	

We included the general terms such as “behavio?ral economic*”, “nudg*”, asymmetric paternalism” and “choice architect*” under intervention. To identify specific key terms to use for specifying the intervention, we consulted current literature on existing classification systems of behavioral economics-informed interventions. Of the various classification systems that have been proposed [27–29], we found the taxonomy proposed by Münscher et al [26] to be appropriate for our review as it classifies interventions into mutually exclusive and exhaustive categories, thereby facilitating inter-study generalizability and knowledge accumulation. Thus, our search terms included for intervention were informed by the 9 subcategories proposed by Münscher et al [26] along with terms identified from our initial exploratory search (Table 1).

### Eligibility criteria

We included primary research articles published in the English language that met the following inclusion criteria: (i) the study design was randomized and controlled or quasi-experimental; (ii) if study was conducted on a mixture of different healthcare providers, the majority were physicians; (iii) physicians of all specialties in all care settings; (iii) a behavioral outcome was objectively measured, as opposed to attitudes or preferences. Studies were excluded if they met the following criteria: (i) the full text could not be obtained; (ii) set in low- or middle-income countries; (iii) targetted patient behavioral change. Healthcare system challenges in low- and middle-income countries such as unstable governance structures, limited resources in service delivery, and limited access to healthcare differ substantially from high-income countries [30]. Thus, to ensure external validity we decided to restrict our review to focus on high-income countries as interventions may affect providers differentially in these different settings.

### Data extraction and analysis

We imported all retrieved studies into Zotero reference management software and removed duplicates. One reviewer (SW) conducted a title screen on all retrieved articles based on the previously described criteria. Two reviewers (SW, NL) then independently screened the abstract and full text of the remaining studies and documented reasons for exclusion. Any disagreements at this stage were resolved by an independent third reviewer (OG).

We developed a standardized data extraction form in Microsoft Excel based on a Cochrane collaboration form and on reporting guidelines for randomized controlled trials in behavioral medicine [31,32]. Data we collected includes study context, research design, intervention and outcome. We piloted the form on 10% of included articles to ensure feasibility, completeness, and consistency of extraction by reviewers, and iteratively refined the form as needed.

We categorized the interventions of included studies using a taxonomy proposed by Münscher, Vetter, & Scheuerle (2016), with interventions classified into three broad categories and nine subcategories (Table 2). Interventions categorized as “Decision Information” focus on the presentation of information relevant to the decision and includes translating information, making information visible, and providing social reference points [26]. While “translating information” aims to change the format or presentation of information while retaining original content, “making information visible” aims to bring previously hidden information to the forefront [26]. Interventions categorized as “Decision Structure” alter the arrangement of the options or the decision-making format. This includes changing choice defaults, changing option-related efforts, changing range or composition of options, and changing option consequences [26]. An example of “changing option-related efforts” in a healthcare setting can be installing hand sanitizers at eye level or at entrances to decrease the effort required. An example of “changing the range or composition of options” in the healthcare setting is having different medication options presented to prescribers either spread horizontally or stacked vertically. Interventions categorized as “Decision Assistance” aims to help decision-makers follow through with their intentions, and includes providing reminders and facilitating commitment [26]. We categorized physician behaviors inductively by reviewing all measured outcomes in included studies.

**Table 2:** Categorization of interventions in included studies based on taxonomy developed by Menscher et al (2016).

Category	Sub-categories	Definition; example
A. Decision Information	A1 Translate information	Change presentation or format (not content) to translate existing information to assist decision-making.
		Ex) Equivalence framing
	A2. Make information visible	Making decision-relevant inaccessible information more apparent or readily available.
		Ex) Feedback reports
	A3. Provide social reference point	Influencing individual’s behavior by illuminating group behavior
		Ex) Refer to opinion leader, referring to social norms
B. Decision Structure	B1. Change choice defaults	Preselected options that leaves decision makers the freedom to select alternatives
	B2. Change option-related effort	Increasing or decreasing the physical or marginal financial effort required associated with choosing an option
		Ex) placing hand sanitizers at eye-level by entrances
	B3. Change range or composition of options	Changing what choices are presented to decision makers to influence the relative attractiveness of options.
		Ex) Decoy options
	B4. Change option consequences	Modifying consequences of decision options by providing “micro-incentives” which would be considered insignificant from a rational choice perspective.
		Ex) Offering participation in a lottery for each day people adhered to medication prescription
C. Decision Assistance	C1. Provide reminders	Providing reminders to overcome limits of inattention and cognitive capacity
	C2. Facilitate commitment	Facilitate commitment to counteract self-control problems and bridge the intention-behavior gap
		Ex) Making a public commitment

Two reviewers extracted and assessed the data independently, and discrepancies were resolved through discussion with a third reviewer or clarification from study authors. We developed a template to query authors via email in the case of missing data. If the study author did not respond to the query, the requested data were treated as missing data.

Included studies were highly heterogenous in the intervention type and implementation, the target population and setting, the outcome measures reported, and the measures used to determine intervention effect. Thus, we conducted a narrative synthesis by comparing results of studies within each intervention category instead of a meta-analysis.

### Quality assessment

Included studies were assessed for methodological quality and risk of bias using the Effective Public Health Practice Project (EPHPP) tool [33]. This tool evaluates the overall quality of a study based on internal validity (study design, confounders, data collection methods) and external validity (sampling, actual participation). Each included study received a score of weak, moderate, or strong.

## Results

**Fig 1:**
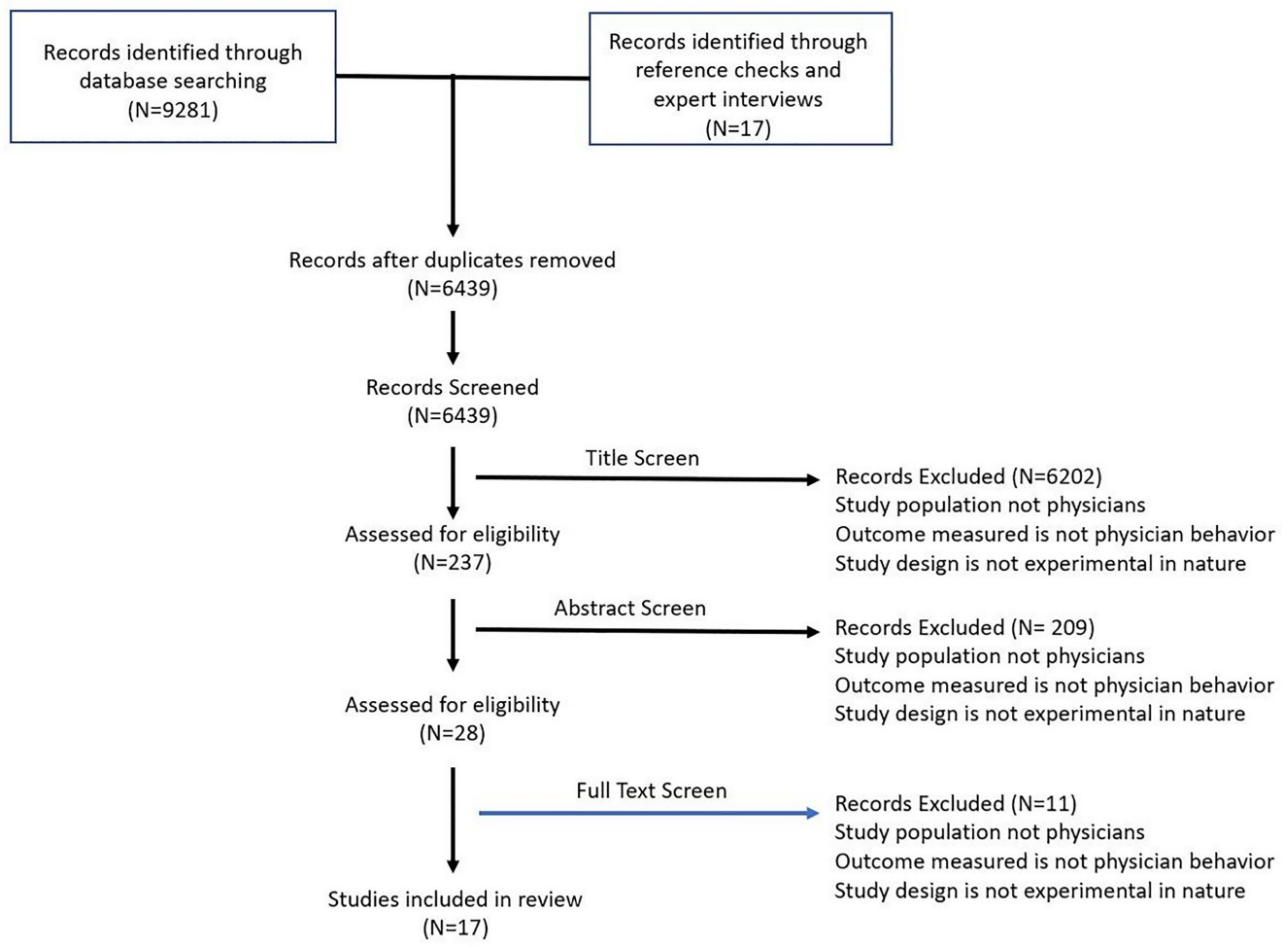
PRISMA diagram of included studies.

Of the 6,439 citations our search returned, we screened 237 abstracts and identified 17 studies involving at least 9,834 healthcare providers that met our prespecified inclusion criteria (Fig 1).

### Description of studies

Most of the studies (n = 14) were conducted in the United States, and in the United Kingdom. Studies were mainly experimental (n = 10), were set in multiple clinics or practices (n = 8), and most were published between 2014 and 2018 (Table 3.), illustrating the development and interest in this field in recent years. Sample size ranged from 14 (clinic-level study) [34] to 5,055 (physician-level study) [35]. Most studies were of general practitioners (n = 13), and in the patient safety and quality domain (n = 7). Most of the included studies were rated as moderate (n = 8) and strong (n = 7).

**Table 3:** Descriptive statistics of included papers investigating the effectiveness of behavioral economics informed interventions on physician behavioral change (n = 17).

Characteristic	No. (%)
**Location**	
United States	14 (82.3)
United Kingdom	3 (17.7)
**Year**	
2014	3 (17.6)
2016	5 (29.4)
2017	1 (5.9)
2018	8 (47.1)
**Setting**	
Multiple hospitals	4 (23.5)
Hospital-wide within 1 hospital	1 (5.9)
Hospital ward or department	3 (17.6)
Multiple clinics or practices	8 (47.1)
Individual clinic or practice	1 (5.9)
**Research design**	
Experimental	10 (58.8)
Quasi-Experimental	7 (41.2)
**Healthcare performance domain**	
Patient safety and quality	7 (41.2)
Clinical disease management	4 (23.5)
Health promotion and disease prevention	2 (11.8)
Cost control	4 (23.5)
**Medical discipline**	
General practice	13 (76.5)
Internal medicine	2 (11.8)
ICU	1 (5.9)
Emergency	1 (5.9)

The 17 included studies investigated 10 distinct interventions belonging to six categories, including changing choice defaults (n = 7), providing social reference points (n = 7), and facilitating commitment (n = 3) (Table 4). Most interventions (n = 13) investigated the effectiveness of one intervention compared to usual care, while three studies simultaneously compared the effects of different interventions separately [36–38] or in combination [39] on one behavioral outcome. Most studies found a significant positive effect on targetted behavior. Only three studies [37,38,40] identified an underlying theory that specifies the mechanism of action for each studied intervention and target behavior. The most common behavioral outcomes studied were prescribing (n = 12) and diagnostic test ordering (n = 4).

**Table 4:** Overview of interventions examined in included studies by intervention category and primary outcome category.

	Prescription		Diagnostic test ordering	Preventive services
	Antibiotics	Other		
**Make Information Visible**	Price transparency feedback [41]	Patient deceased letter [42] Price transparency feedback [41]		
**Provide Social Reference Point**	Peer comparison [37,43] Social norm feedback [44]	Peer comparison [35,38,39]	Social comparison [45]	
**Change Choice Defaults**		Active choice [39] Default [40,46] Order set [47]	Order set [48]	Active choice [49,50]
**Change Option Related Effort**	Accountable justification [37,43]			
**Change Range**	Suggested Alternatives [37,43]			
**Facilitate Commitment**	Commitment poster [34,51]		Precommitment [51]	

### Intervention outcomes

#### Change choice defaults

Interventions in this category can be further subdivided into prompted choice which includes active choice and no-action defaults which includes default and order set (Table 4). Active choice is an intervention where decision makers are prompted to choose without a pre-selected option within the choice environment. Default is presented as a pre-selected option that freely allow decision makers to choose another option. Order sets are a prepackaged group of orders that apply to a specific diagnosis and is used in healthcare to standardize patient care and reduce errors. Studies in this category were of strong [39,40,48], moderate [46,49,50] and weak [47] quality and targeted behaviors from all categories (Table 5).

**Table 5:** Characteristics of included studies (n = 17).

Study author; year; Country *(Study ID)*	Research Design, Quality Assessment	Sample	Setting	Intervention Description	Duration; delivery; Target Group	Primary Outcome; Comparison	Effect Size (95% CI)
Patel et al; 2018; United States (3)	Cluster randomized controlled trial; Strong	96 PCPs: 32 PCPs in usual care 32 PCPs in active choice 32 PCPs in active choice with peer comparison	32 clinics at the University of Pennsylvania Health System	Active Choice; PCPs are emailed dashboard that lists patients eligible for statin prescription and must select whether to prescribe statin or not (justification required)	2-month; Dashboard active for 2 months, 2 email reminders were provided; Individual general practitioners	Change in percentage of eligible patients prescribed a statin. Compared to usual care	Adjusted difference in percentage points: 4.1% (CI: -0.8 to 13.1)
				Active Choice + Peer Comparison; PCPs are also provided feedback on their baseline statin prescribing rates prior to entry in the trial compared with their peers.			Adjusted difference in percentage points: 5.8% (CI: 0.9 to 13.5)
Patel; 2016; United States (4)	Quasi-experimental; Moderate	Not reported	3 internal medicine practices at the University of Pennsylvania Health System (within 0.3 miles apart)	Active Choice; Physician is prompted to “accept” or “cancel” a test order for colonoscopy or mammography as appropriate.	1 year, Embedded in EMR; Individual specialists	Percentage of eligible patients that had appropriate test ordered by physician; Compared to control	Difference in difference estimator: 11.8% (CI: 8.9 to15.6)
		Colonoscopy: 2224 patients in intervention 2753 patients in control 1 2583 patients in control 2					
		Mammography: 2929 patients in intervention 2974 patients in control 1 2434 patients in control 2					Difference in difference estimator: 12.4% (CI: 8.7 to 16.2)
Patel et al; 2017; United States (5)	Quasi-experimental; Moderate	Not reported 17,249 patients in intervention 28,686 patients in control	3 internal medicine practices at the University of Pennsylvania Health System (within 0.3 miles apart)	Active Choice; Physician is prompted to actively choose to “accept” or “cancel” the influenza order for eligible patients during visit.	1 year, Embedded in EMR; Individual specialists	Percentage of patients eligible for the influenza vaccine who had an order for it on the day of the clinic visit; Compared to control	Difference in difference estimator: 6.6% (CI: 5.1 to 8.1)
Persell et al; 2016; United States; Strong (6)	Randomized Controlled Trial; Strong	27 internists and 1 nurse practitioner	An adult primary care practice affiliated with an academic medical center in Chicago	Accountable Justification; Physician received alert when prescribing antibiotics that summarizes guidelines and prompt to enter justification for prescription (made available on EHR)	1 year; Embedded in EMR; Individual general practitioners	Rate of guideline concordant oral antibiotic prescribing during the eligible study; Compared to baseline	Odds Ratio OR = 0.98 (CI: 0.42 to 2.29)
				Suggested Alternatives; Physician presented with order set containing non-antibiotic prescription, non-prescription medication choices and patient educational material			Odds Ratio OR = 0.68 (CI: 0.29 to 1.58)
				Peer Comparison; Clinicians receive monthly performance feedback that include their antibiotic prescribing rates and that of colleagues at the lowest 10^th^ percentile.			Odds Ratio OR = 0.45 (CI: 0.18 to 1.11)
Meeker et al; 2014; United States (10)	Randomized Controlled Trial; Moderate	11 physicians and 3 nurse practitioners	5 Los Angeles community clinics	Public Commitment; A poster-sized letter signed by clinicians and posted in their examination rooms indicating their commitment to reducing inappropriate antibiotic use.	3 months; Poster on wall; Individual physicians	Relative frequency of patients receiving antibiotic prescription for antibiotic-inappropriate ARI diagnoses.	Difference in difference estimator: -19.7% (CI: -33.4 to -5.8)
Meeker et al; 2016; United States (12)	Randomized Controlled Trial; Strong	248 clinicians	49 primary care practices from 3 health systems using 3 different electronic health records in 2 geographically distinct regions: Massachusetts and Southern California	Suggested Alternatives; Clinicians prescribing antibiotics receive a pop-up screen indicating that antibiotics is inappropriate and suggests a list of alternatives.	18 months; Embedded in EMR; Individual physicians	Antibiotic prescribing rate for antibiotic-inappropriate acute respiratory tract infection visits and no concomitant reason for antibiotic prescribing	Difference in difference estimator: -5% (CI: -7.8 to 0.1)
				Accountable Justification; Clinicians prescribing antibiotics receive a prompt asking each clinician to justify, in a free text response, his or her treatment decision.			Difference in difference estimator: -7% (CI: -9.1 to -2.9)
				Peer Comparison; Clinicians with the lowest inappropriate antibiotic prescribing rates were told that they were “Top Performers,” and others were informed of their standing.			Difference in difference estimator: -5.2% (CI: -6.9 to -1.6)
Bourdeaux et al; 2014; United Kingdom (14)	Quasi-experimental; Weak	Approximately 20 clinicians	Mixed medical and surgical ICU at University Hospital Bristol NHS Foundation Trust. Tertiary ICU with over 1200 admissions/year	Order set design; Admitting doctors can choose to use a prescribing template with some commonly used drugs and fluids prescribed. They populate the final electronic drug chart by selecting which drugs from the template not to give.	1 time; Embedded in EMR; Individual specialists	Number of ventilated patients prescribed chlorhexidine 4 times per day per number of ventilated patients on the ward	Percentage change: 35.1%
							p<0.001
						Number of patients given HES per number of patients on the ward.	Percentage change: -51.0%
							p<0.001
Patel et al; 2015; United States (15)	Quasi-experimental; Strong	255 physicians: 204 Intervention 51 Control	2 ambulatory clinics in the Division of General Internal Medicine and 2 ambulatory clinics in the Department of Family Medicine	Default; When the provider searched from brand-name medication, the results listed only dosing options for generic-equivalent medications.	1 time; Embedded in EMR; Individual specialists	Monthly prescribing trends of generic medication equivalents in the pre and post intervention periods. Compared to control	Difference in difference estimators
							Beta blockers: 10.5% (CI: 5.8 to 15.2)
							Statins: 4% (CI: 0.4 to 7.6)
							Proton-pump inhibitors: 2.1% (CI: -3.7 to 8.0)
Munigala et al; 2018; United States (17)	Quasi-experimental; Strong	Not reported	Emergency department at hospital	Order set design; Removing other urine test orders and retaining only “urinalysis with reflex to microscopy.”	1 time; Embedded in EMR; Individual specialists	Primary outcome measure not directly specified. Daily urine culture rate per 1000 ED visits. Pre vs. post	Percentage change: -46.6% (CI: -66.2 to -15.6)
Sacarny et al; 2018; United States (19)	Randomized Controlled Trial; Weak	5055 physicians: 2528 control 2527 intervention	Nationwide (prescribers of quetiapine with a specialty of general practice, family medicine, or internal medicine)	Social Comparison Feedback; A mailed peer comparison letter indicating that the prescriber’s quetiapine prescribing was under review and was extremely high relative to the within-state peers.	9 months; Letter sent; Individual general practitioners	Cumulative total number quetiapine days supplied by physician in the 9 months after the intervention start; Compared to control	Percentage difference: -11.1% (CI: -13.1 to -9.2)
Hallsworth et al; 2016; United Kingdom (20)	Randomized Controlled Trial; Moderate	1581 general practitioners: 790 control 791 intervention	General practitioners that practice in England that were in the top 20% for antibiotic prescription in the NHS local area team	Social Comparison Feedback; Clinicians receive a letter detailing how the recipient’s practice’s prescribing rate compared with other practices in the local area.	1 time; Letter sent; General practitioner clinic	Rate of antibiotic items dispensed per 1000 population; Compared to control	Incidence rate ratio 0.967 (CI: 0.957 to 0.977)
							Estimated 73,406 fewer antibiotics prescribed
Doctor et al; 2018; United States (22)	Randomized Controlled Trial; Moderate	826 physicians: 438 control 388 intervention	All clinicians and allied health professionals with scheduled drug prescribing privileges in California were targetted	Feedback; Provider who wrote a drug prescription that resulted in a fatal scheduled drug overdose receives a signed letter notifying them of a death in their practice	1 time; Letter sent; Individual physicians	Adjusted daily average change in milligram morphine equivalents (MME) dispensed per prescriber; Compared to control	Change in MME: -6.9 (CI: -13.1 to -1.0)
Chiu et al; 2018; United States (23)	Quasi-experimental; Moderate	Not reported	3 hospitals that performed most surgical procedures	Default; The default number of opioids prescribed was changed on the EMR.	1 time; Embedded in EMR; Individual physicians	1. Change in median number of opioid pills per prescription	Change in number of pills per prescription
		2910 operations: 1447 pre 1463 post					-5.22 (CI: -6.12 to -4.32)
						2. Total dose of opioid prescribed per order.	Change in total dose -34.41 MME (CI: -41.36 to -27.47 MME)
Langley et al; 2018; United Kingdom (25)	Quasi-experimental; Weak	Not reported	Royal Derby Hospital is a busy acute medical hospital that admitted 140,960 individuals in 2014	Cost Feedback; The cost of the drug prescribed is made available to the prescribing clinician	2 years; Embedded in EMR; Individual physicians	Weekly cost for antibiotics prescription in the intervention period compared to baseline costs.	Mean weekly expenditure on antibiotics per patient: -£3.75 (CI -6.52 to -0.98)
		Mean number of patients prescribed antibiotics and inhalers were 428 and 55 individuals per week, respectively.					However, slowly increased subsequently by £0.10/ week (CI 0.02 to -0.18)
						Weekly cost for inhaled corticosteroids prescription in the intervention period compared to baseline costs.	Mean weekly expenditure -£0.03 pounds(CI: -0.06 to -0.01)
Ryskina et al; 2018; United States (26)	Randomized Controlled Trial; Moderate	114 physicians 39 intervention 34 control 41 both intervention and control	6 general medicine teams at the hospital of the University of Pennsylvania	Social Comparison Feedback: Email summarizing provider’s routine lab ordering vs. the service average for the prior week, l	6 months; Embedded in EMR; Physician team	Count of routine laboratory orders placed by each physician per patient-day.	Adjusted difference -0.14 (CI: -0.56 to 0.27)
Kullgren et al; 2018; United States (27)	Randomized Controlled Trial; Moderate	45 physicians	6 primary care clinics of Integrated Healthcare Associates (IHA), a multispecialty group practice in South East Michigan	Precommitment; Clinicians receive point-of-care reminders of their precommitment attached to a patient education handout, as well as weekly emails with links to resources to improve communication with patients about low-value services.	1–6 months; Paper based; Individual general practitioner	Change in percentage of visits with orders for potentially low-value service; Pre vs post	Percentage change:Low back pain: -1.2% (CI: -2.0 to -0.5)
							Headaches: 0.7% (CI: -0.7 to 2.1)
							Acute sinusitis: -3.4% (CI: -8.2 to 1.4)
Scarany et al; 2016; United States (28)	Randomized controlled Trial; Strong	1518 physicians	Nationwide (targetted high prescribers of Schedule II prescriptions)	Social comparison feedback; High prescribers were sent 1 letter indicating their prescribing rates of Schedule 2 controlled substances were far higher than their peers (same specialty of same state).	One time; Paper based; Individual physicians	Change in Schedule II prescription fills (adjusted for days’ supply) over the 90 days following the mailing.	3.5 fills (CI: -6.35, 13.40)

One US-based research group examined the effectiveness of active choice in three separate studies, and found positive impacts on increasing the rate of cancer screening, influenza vaccination orders, and on increasing guideline concordant statin prescribing [39,49,50]. Active choice was implemented as a prompt delivered prior to the patient’s clinical visit, alerting the decision-maker of the patient’s eligibility for the desired action—statin prescription, cancer screening, and influenza vaccination. Active choice intervention increased the rate of cancer screening tests [49] and flu vaccination [50] ordered. For statin prescribing, the positive impact was only noted when implemented in conjunction with peer-comparison intervention but not as a stand-alone intervention [39]. Researchers noted the importance of integrating the intervention into the clinical workflow as a contributing factor to effectiveness.

No-action default was examined in four studies—two in defaults and two in order set redesign, and all studies found these interventions to be effective in changing prescribing practices (Table 5). The studies varied in their implementation. One study examined the effectiveness of a lowered default opioid pill count set for postoperative analgesia and provided an education session for physicians as part of its implementation [46]. Another study examined the use of defaults to decrease medication costs by changing the electronic prescribing system to only initially display generics, instead of displaying them alongside the brand name drugs for which prescribers searched. Changes in defaults were associated with an increase in generics prescribing, though not for all medications studied (statins, proton pump inhibitors and beta blockers) and not among resident physicians [40]. Authors attributed the insignificant increase in generic proton pump inhibitor prescribing to its availability as an over the counter drug, so patients may already have a preference [40]. Additionally, authors postulated that the smaller effect of default setting on resident physician prescribing was due their higher baseline prescribing of generics [40]. The two studies investigating the use of order sets took place in busy clinical settings and found positive impacts. One study aimed to decrease unnecessary urine test orders and had an education session with physicians regarding the removal of the urine test option [48]. The other study aimed to improve guideline concordant care of mechanically ventilated patients in intensive care units by implementing evidence-based order sets with prepopulated medication orders [47].

#### Provide social reference point

Providing a social reference point for decision-makers was the second most studied intervention (n = 7) (Table 4). Interventions in this group provided physicians with feedback on their performance relative to their peers on select quality indicators. We included in this group studies using interventions termed peer comparison, social comparison, and social norm feedback. All studies used a randomized controlled design and were of strong [38,39,43], moderate [34,44,52] and weak [35] quality (Table 5). Studies examined interventions’ impact on antibiotic prescribing behavior [37,43,44], statin prescribing [39], antipsychotic prescribing [35], controlled substances prescribing [38] and laboratory test ordering [52].

Four of the seven studies found peer comparison feedback to positively impact prescribing behavior [35,43,44], one of which was implemented in conjunction with active choice [39]. While Hallsworth et al found social comparison to have a significant positive impact on decreasing inappropriate antibiotic prescribing, it was not possible to disentangle the independent effects of social comparison, high profile messenger, and recommended actions in the single letter that was sent as the intervention [44].

Of the three studies that did not find this intervention to be effective, two were implemented as pilots [35,37] with larger scale randomized trials implemented later that did show intervention effectiveness [35,43]. Persell et al found peer comparison to be effective at reducing antibiotics prescribing for all acute respiratory illnesses but not specifically antibiotic-inappropriate diagnoses in the pilot study [37]. However, in a later and larger randomized trial, the same research group found peer comparison to be effective at reducing inappropriate antibiotic prescribing [43]. Authors attributed the null effects of peer comparison in the pilot to the small sample size and to potential contamination which were both addressed in the later study by increased sample and block randomization. In the other set of studies, Sacarny et al found peer comparison letters to increase the inappropriate prescription of controlled substances among high-prescribers, though the effect was not statistically significant [38]. In a subsequent study, peer comparison was found to be effective in reducing antipsychotic prescribing among high-prescribers [35]. Authors attributed the positive findings in the latter study to the increased intensity of the interventions (peer comparison letter sent with two subsequent reminders), refined target population using more recent data and stronger wording that triggered action [35]. The last study to find null effect of peer comparison was designed to decrease unnecessary laboratory testing [52]. As potential explanations for the null findings, authors noted a lack of engagement among study participants with the peer comparison dashboard, cross-contamination, and an undifferentiated target group [52]. Additionally, this study targetted interns and residents during the two week service block and utilized the entire medical team’s prescribing data rather than individual prescribing data [52].

#### Make information visible

We identified two studies [41,42,53] that aimed to bring often invisible behavioral consequences to the attention of decision makers through feedback provision (Table 4). Study quality was rated as moderate [42], and weak [41] (Table 5). Providing feedback to change physician behavior is an extensively researched field [54]. Included studies examined the effectiveness of feedback on reducing opioid prescribing [42], and spending on antibiotics prescriptions [41].

Two studies found the intervention positively changed the target behavior [41,42]. Implementation and the feedback content differed drastically across studies. Doctor et al sent one letter to prescribers notifying them of their patients’ deaths due to opioid overdose and observed a significant decrease (9.7%, 95%CI 6.2 to 13.2) in milligram morphine equivalents filled three months after the intervention [42]. Lastly, Langley et al found that having the drug cost continuously displayed on the electronic prescribing system reduced the mean weekly expenditure of antibiotic prescriptions by £3.75 (95%CI –6.52 to –0.98) per patient at the start, however cost increased steadily by £0.10 per week (95%CI 0.02 to 0.18) afterwards [41] during the 12 week study period.

#### Change option-related effort and change range or composition of options

We identified two studies (pilot and follow-up) conducted by one US-based research group that concurrently examined effectiveness of interventions grouped into these two categories aimed at decreasing antibiotic prescribing (Table 4). Accountable justification is an intervention whereby clinicians receive alerts via the electronic prescribing platform containing prescribing guidelines and prompting a justification for the antibiotic prescription. Suggested alternative is an intervention whereby alternative options such as non-antibiotic prescriptions, non-prescription medications, and patient-oriented educational materials are presented in the electronic prescribing system when an antibiotic is entered.

Both pilot [37] and follow up studies [43] concurrently examined the efficacy of accountable justification, suggested alternatives, and peer comparison in reducing antibiotic prescribing (Table 5). Participants in both studies received an education module on guideline-concordant antibiotic prescription prior to intervention. While the pilot study [37] did not find either of the interventions to significantly reduce antibiotic prescribing (OR = 0.98, 95%CI: 0.42–2.29), the follow-up, a higher powered study using same methods [43] did significantly reduce inappropriate antibiotic prescribing (difference in difference -7.0%, 95%CI: -9.1 to -2.9).

#### Facilitate commitment

Facilitating commitment bridges the intention-behavior gap by counteracting self-control problems making individuals more likely to follow through with actions [26]. Effectiveness of commitment was examined in reducing antibiotic prescriptions and unnecessary diagnostic imaging in two studies, both of moderate-quality [34,51] (Table 5). One study explored the impact of a public commitment poster—written in accessible language, signed by the physicians, and posted in the examination room for three months. This was effective in reducing the rate of inappropriate antibiotic prescribing up to 12 months at follow-up. Another study found precommitment to have a small but statistically significant effect on reducing low-value imaging for lower back pain. In this study, precommitment was reinforced via a paper-based reminder provided to physicians at the point of clinical encounter. However, the effect was not sustained after 3 months [51].

## Discussion

Our systematic review including seventeen articles investigating the effectiveness of behavioral-economics informed interventions on changing physician behavior found the two most studied interventions—changing default settings and providing social reference points, were consistently effective in behavioral change. A large volume of research exists examining the effectiveness of audit and feedback and reminder provision on physician behavior, and is reviewed extensively elsewhere [2,55]. However, few studies in these two intervention categories were included in our review as most were not used to address a behavioral change with an underlying cognitive bias. The most common behavioral outcome targeted was prescribing behavior, with most of these studies examining decreasing inappropriate antibiotic prescribing. To our knowledge, this is the first systematic review of the use and effectiveness of behavioral economics-informed interventions on physician behavior.

Changing default settings is a common approach, likely due to its relative ease of implementation and effectiveness in different contexts [56]. In our review, we included both prompted choice and no action default in this intervention category. We noted differences in implementation of active choice between included studies which may be associated with the intervention effectiveness. Specifically, we found that studies targeting both clinicians and medical assistants found active choice to be effective as a standalone intervention, but not when targeting only clinicians. This was further explored in a later study by the same US-based research group where they found active choice to have an even larger effect on increasing clinician ordering of cancer screening tests when targeting only medical assistant [57]. Researchers noted the importance of considering relieving physician burden and particularly alert fatigue among physicians when implementing active choice interventions.

All included studies investigating no-action default found a significant positive effect on behavioral change. This finding is consistent with that from a recent meta-analysis of 58 default intervention studies that found sizeable and robust effects [56]. However, there was substantial variation in the effect size across studies which authors noted may be due to an imperfect understanding of the mechanisms of action [56]. Indeed, only one [40] of four studies that examined the effectiveness of no-action defaults referenced the theorized mechanism of effect.

A theoretical framework proposed by Dinner et al outlines three possible mechanisms of how default interventions influence behavior—endowment, endorsement and effort [58]. It postulates that individuals are more likely to choose a preselected option because: (1) alternatives are evaluated in reference to the preselected option which is already endowed (2) preselected option is viewed as endorsement from the choice architect (3) less effort is exerted when a preselected option is chosen [58]. Literature suggests that effectiveness may increase when more mechanisms of action are activated [56]. Additionally, increasing evidence suggests that disclosure of a default may further increase the intervention effectiveness [59,60]. It is theorized that transparent disclosures can foster positive inferences such as trust and credibility of the default intervention implementor thereby increasing compliance, which aligns with the postulated endorsement mechanism. This may have contributed to the success of two included studies that briefed physicians on changes made to an existing order set [48] and implementation of a default change in opioid prescribing prior to implementation [46].

Social comparison was the second most frequently studied intervention in our review and generally resulted in significant positive effects on changing prescribing behavior [35,39,43,44]. We found notable differences in how this intervention was implemented across studies. When comparing studies that showed a positive significant effect [35,43,44] to those that did not find a significant effect [40,52], we did not find consistent explanatory characteristics such as mode of communication (email vs paper), frequency (one time vs recurring) and duration of intervention.

Providing a social reference point is theorized to change behavior by engaging individuals in “upward comparison”, with individuals evaluating their own performance against superior performers [61]. While all studies compared participants with better performance, the choice of comparator differed. Persell et al argued that comparing individuals with high performers sustains high performance; their study compares antibiotic prescribing rates of all participating physicians with rates of the top 10 percentile [37]. In contrast, Patel et al segmented participating physicians into three groups and provided different comparators [39]. While both interventions showed a significant positive effect on changing prescribing rates, research suggests that individuals engage in upward social comparison with the goal of self-improvement when they perceive the comparator to be similar to oneself [61]. This supports the comparison to similar performing others in the latter study [39].

Based on our findings, default interventions seem most effective when targeting behaviors contained within an electronic system such as prescription or test orders due to its simplicity in implementation and workflow integration. It may also be helpful to be transparent when implementing default interventions research has shown disclosure to engender trust [59,60]. For organizations engaged in a culture of feedback, we found incorporating social reference points for individuals to be effective in guiding individuals to evaluate their own performance and engage in upward comparison. Among included studies, we found studies that employed a moving target, utilized up-to-date data, and chose a comparator that target individual can identify with saw positive impact.

### Limitations

Our study has important limitations. First, no consensus exists on which interventions are informed by behavioral economics and no common terminology is utilized in reference to the same concepts. While we were comprehensive in our search by incorporating not only terms from our chosen taxonomy but also known related concepts, it is conceivable that relevant studies may inadvertently be overlooked. In addition, this further limits our ability to compare and contrast our findings of intervention effectiveness with other fields. Second, as most studies (n = 13) did not include a follow-up period of more than 6 months, it was not possible for us to determine the intervention’s sustainability and longer-term effects. Thus, conclusions from this review should be interpreted as effectiveness for behavioral change and should not be confused with behavioral maintenance. Third, this review is heavily weighted by evidence from general practitioners, thus indicating a need for studies examining the applicability of these types of interventions among specialists due to differences in practice environments. Finally, although we have selected a taxonomy with mutually exclusive categories, categorizing interventions is difficult when intervention theory or mechanism of action is not described, as was the case for most of the included studies.

### Recommendation for future research

Researchers should aim to design theory-informed intervention while considering the underlying barrier to behavioral change. This can not only increase the potential for intervention effectiveness, but also improve our understanding of the intervention’s mechanism of action. Further, outcomes should be examined over a longer follow-up period. This not only sheds light on intervention sustainability but also potential negative impacts. Finally, a common terminology for the interventions informed by behavioral economics should be adopted to improve evidence accumulation. Although not without its limitations, we recommend the use of Münscher et al’s taxonomy [26].

## Conclusion

We find that changing default settings and providing social reference points were the most commonly studied behavioral economics-informed interventions employed to change physician behavior. Both classes of interventions were generally effective in positively changing physician behavior, particularly in prescribing.

## Supporting information

S1 Table Overview of intervention type and study results. (DOCX)

S1 Checklist Prisma checklist. (DOC)
