# Supplementary material for: The effectiveness of behavioral economics-informed interventions on physician behavioral change: A systematic literature review
Source: PLoS One. 2020 Jun 4;15(6):e0234149. doi: 10.1371/journal.pone.0234149 (PMC7272062; doi:10.1371/journal.pone.0234149)
Supplement: S1 Table — (DOCX) [file pone.0234149.s001.docx]

**S1. Table.** Overview of intervention type and study results

Overview of intervention type and study results

| **Intervention Type** | **Intervention** | **Target Outcome** | **Behaviour Category** | | | |  |
| --- | --- | --- | --- | --- | --- | --- | --- |
|  |  |  | **Prescribing** | | **Diagnostic test ordering** | **Prevention** | |
|  |  |  | **Antibiotics** | **Other** |  |  |  |
| **Make information visible** | Patient death letter (50) | ↓ inappropriate opioid prescription |  | +* |  |  | |
|  | Cost feedback (51) | ↓ antibiotics prescription | +* |  |  |  | |
|  | Cost feedback (51) | ↓ corticosteroids prescription |  | + |  |  | |
| **Provide social reference point** | Peer comparison (34) | ↓ inappropriate antibiotic prescription | + |  |  |  | |
|  | Peer comparison (47) | ↓ inappropriate antibiotic prescription | +* |  |  |  | |
|  | Social norm feedback (48) | ↓ inappropriate antibiotic prescription | +* |  |  |  | |
|  | Peer comparison (33) | ↓ inappropriate antipsychotic prescription |  | +* |  |  | |
|  | Social comparison (49) | ↓ unnecessary lab test order |  |  | + |  | |
|  | Social comparison (37) | ↑ inappropriate controlled drug prescription |  | - |  |  | |
| **Change choice defaults** | Active choice (38) | ↑statin prescribing |  | + |  |  | |
|  | Active choice (61) | ↑ colonoscopy order |  |  |  | +* | |
|  | Active choice (61) | ↑ mammography order |  |  |  | +* | |
|  | Active choice (62) | ↑ influenza vaccination order |  |  |  | +* | |
|  | Order set design (46) | ↑ use of chlorhexidine for ventilated patients |  | +* |  |  | |
|  | Order set design (46) | ↓ use of Hydroxyethyl starch in IV |  | +* |  |  | |
|  | Order set design (43) | ↓unnecessary urine tests |  | +* |  |  | |
|  | Default (39) | ↑ prescription of beta blocker generics | +* |  |  |  | |
|  | Default (39) | ↑ prescription of statin generics | +* |  |  |  | |
|  | Default (39) | ↑ prescription of proton-pump generics | + |  |  |  | |
|  | Default (40) | ↓ inappropriate opioid prescription |  | +* |  |  | |
| **Change option-related effort** | Accountable justification (34) | ↓ inappropriate antibiotic prescription | + |  |  |  | |
|  | Accountable justification (47) | ↓ inappropriate antibiotic prescription | +* |  |  |  | |
| **Change range or composition of options** | Suggested alternatives (34) | ↓inappropriate antibiotic prescription | + |  |  |  | |
|  | Suggested alternatives (47) | ↓ inappropriate antibiotic prescription | + |  |  |  | |
| **Facilitate commitment** | Public commitment poster (32) | ↓ inappropriate antibiotic prescription | +* |  |  |  | |
|  | Precommitment (54) | ↓ order for lumbar spine Xray , CT or MRI |  |  | +* |  | |
|  | Precommitment (54) | ↓ order for head CT or MRI |  |  | - |  | |
|  | Precommitment (54) | ↓ inappropriate antibiotic prescription |  |  | + |  | |

*indicates a statistically significant difference between the control and intervention group at the 0.05 level

+ indicates a positive change according to the intervention intended directionality

- indicates a negative change according to the intervention intended directionality
